# Effect of polarization on HIV-1protease and fluoro-substituted inhibitors binding energies by large scale molecular dynamics simulations

**DOI:** 10.1038/srep42223

**Published:** 2017-02-03

**Authors:** Li L. Duan, T. Zhu, Yu C. Li, Qing G. Zhang, John Z. H. Zhang

**Affiliations:** 1 School of Physics and Electronics, Shandong Normal University, Jinan 250014, China; 2 Department of Chemistry, East China Normal University, Shanghai 200062, China; 3 NYU-ECNU Center for Computational Chemistry at NYU Shanghai, Shanghai 200062, China

## Abstract

Molecular dynamics simulations in explicit water are carried out to study the binding of six inhibitors to HIV-1 protease (PR) for up to 700 ns using the standard AMBER force field and polarized protein-specific charge (PPC). PPC is derived from quantum mechanical calculation for protein in solution and therefore it includes electronic polarization effect. Our results show that in all six systems, the bridging water W301 drifts away from the binding pocket in AMBER simulation. However, it is very stable in all six complexes systems using PPC. Especially, intra-protease, protease-inhibitor hydrogen bonds are dynamic stabilized in MD simulation. The computed binding free energies of six complexes have a significantly linear correlation with those experiment values and the correlation coefficient is found to be 0.91 in PPC simulation. However, the result from AMBER simulation shows a weaker correlation with the correlation coefficient of −0.51 due to the lack of polarization effect. Detailed binding interactions of W301, inhibitors with PR are further analyzed and discussed. The present study provides important information to quantitative understanding the interaction mechanism of PR-inhibitor and PR-W301 and these data also emphasizes the importance of both the electronic polarization and the bridging water molecule in predicting precisely binding affinities.

Since the beginning of the acquired immune deficiency syndrome (AIDS) pandemic, approximately 78 million people worldwide have been infected and close to 39 million have died of AIDS-related causes. In 2015, it resulted in about 1.1 million people deaths. AIDS is caused by the retrovirus human immunodeficiency virus (HIV) which attacks the human immune system and leaves the body vulnerable to a variety of diseases. HIV type 1 protease (PR) is the essential step in the life cycle of HIV by cleaving the Gag and Gag-pol nonfunctional polypeptides into mature and infectious HIV viral particles. Without the effective of the HIV-1 protease, the viral particles are still non-infectious^1^,^2^. So PR is one of the primary drug targets for anti-AIDS therapy.

PR is a homodimeric enzyme composed of two identical 99 amino acid monomers. Each monomer contains one small α-helix and two twisted antiparallel β-sheets. The active site which comprises two catalytic triplets (ASP25 of A chain/ASP25′ of B chain, THR26/THR26′, GLY27/GLY27′) is located at the dimer interface. The flap regions of the PR (residues 43–58, 43′–58′) are positioned over the active site, and they form two β hairpin structures. The two mobile flaps allow the substrate to enter or leave the substrate-binding cavity. They are in the closed state when the substrate is bound, which, however, shift to a semi open state in the unbound form of PR^3^,^4^,^5^,^6^. The crystal water molecule W301 has an important role in the opening and closing of the flaps as well as increasing the affinity between protein and inhibitor. It is observed in almost all PR and inhibitor complex and forms four hydrogen bonds with inhibitor and the residue Ile50/Ile50′, this bridges the flaps and inhibitor^7^.

Currently, there are ten PR inhibitors approved by the Food and Drug (FDA). They are saquinavir (SQV), ritonavir (RTV), indinavir (IDV), nelfinavir (NFV), amprenavir (APV), lopinavir (LPV), atazanavir (ATV), tipranavir (TPV), fosamprenavir (FPV, prodrug of amprenavir) and darunavir (DRV)^8^,^9^,^10^,^11^,^12^. Several more inhibitors are in late stages of clinical development. However, the current major problem is the evolvement of viral resistance which reduces the binding affinities between PR and inhibitors, so there is still an ongoing need for the development of novel inhibitors for PR^13^,^14^,^15^.

Understanding the interaction mechanisms between PR and inhibitor can provide useful information for accelerating the development of novel PR inhibitors with better potency. Many previous studies have used molecular dynamics (MD) simulation to investigate theoretically the interaction and the binding affinity of these inhibitors^16^,^17^,^18^,^19^,^20^,^21^,^22^. Usually, standard force fields such as AMBER, CHARMM, etc. have been widely used to study the structural and dynamical properties of biomolecules^23^,^24^,^25^,^26^. However, there are fundamental limitations in their applications^27^. Especially, they may fail to give accurate representation of the local electrostatic environment in the binding cavity and the variation of the environment in the active site on binding different inhibitors due to the lack of electronic polarization, especially in long time MD simulation studies^28^. The incorporation of polarization effects into the force field can improve the quality of force fields^29^,^30^,^31^,^32^. It may provide an accurate and reliable description about the binding of inhibitor-PR in which the active site is mainly made up of polar amino acid residues. Therefore, in this paper, we preform extensive MD simulation for six inhibitors and PR complexes in explicit water for up to 700 ns to study the binding affinities using two slightly different polarized protein-specific charge (PPC)^29^ schemes. In addition, MD simulation is also carried out using the standard AMBER force field (AMBER12SB). PPC is derived from the first-principal quantum solvation calculation of entire protein and ligand complex in solution by using molecular fractionation with conjugate caps (MFCC) approach^33^ incorporation the Poisson-Boltzmann (PB) solvation model. By solving the PB equation, the induced charges on solute and solvent interface are obtained to mimic the polarization effect from the solvent molecules. Partial atomic charges of protein and ligand are derived iteratively through DFT calculation in which the induced charges are taken as background charges. The solute and solvent polarize each other until convergence is reached and it is usually in five iterations. Hence, with the implementation of PPC model, the polarization effect of intra-protein, protein-ligand, solvation are all effectively included in the quantum mechanical calculation, so PPC should give a more accurate description about the electrostatic environment than traditional force fields. The advantage of PPC over traditional AMBER force field has been demonstrated in several recent studies^34^,^35^,^36^,^37^,^38^,^39^,^40^,^41^,^42^,^43^,^44^,^45^,^46^,^47^,^48^,^49^,^50^. The previous success of PPC in simulating protein structure and function gives us the confidence that it can properly describe the binding interaction between PR and inhibitors at quantum mechanical level.

The work presented here is the first to implement the polarization effects into long MD simulation to explore the interaction dynamics between six similar inhibitors (BEB, BED, BE3, BE4, BE5 and BE6) and PR. The structures of six inhibitors are shown in Fig. 1(A). BEB is a synthetical C_2_-symmetric diol-based inhibitor^51^. BED and BE3 are the monofluoro-substituted in the 2- (ortho) and 3- (meta) positions of the P1/P1′ benzyloxy side groups inhibitors respectively. BE4, BE5, BE6 are the difluoro-substituted in the 2, 4-, 2, 3-, and 2, 5-positions of the P1/P1′ benzyloxy side groups inhibitors respectively. All six inhibitors with the hydroxyls of the chiral center in staggered and gauche positions interact with the catalytic aspartates ASP25 by short distance hydrogen bond^52^. The crystal W301 is observed in all complexes which bridges the main-chain residues Ile50/Ile50′ to the carbonyls of the inhibitors and the hydrogen bonds network in the initial structure of PR-BEB complex are shown in Fig. 1.

Detailed binding free energies between inhibitors and specific residues of PR and the dynamics properties of the complexes are analyzed and discussed. The results of MD simulation using PPC are explicitly compared with those obtained from the standard AMBER force field. Although FEP(free energy perturbation)^53^ and TI (thermodynamics integration)^54^ methods are more accurate for calculation the binding free energy, they are both prohibitively expensive and are restricted to closely related chemical structures of small molecules. Thus, MM/PBSA method is used to compute the free energies of six inhibitors binding to PR under PPC and AMBER respectively, in order to explicitly investigate the influence of polarization.

## Theoretical Methods

We performed up to 700 ns MD simulations for six systems using the AMBER12SB force field, PPC, and slightly different version of PPC (denotes PPC1) respectively. The initial crystal structure of the PR and six inhibitors complexes are obtained from the Protein Data Bank (PDB entries: 1EBY, 1EC0, 1W5V, 1W5W, 1W5X and 1W5Y for inhibitors BEB, BED, BE3, BE4, BE5 and BE6 respectively) and they are taken as the starting structure for dynamic simulations. Due to the importance of the protonation states of the two catalytic aspartic acids of PR, the OD2 of ASP25 in A chain is protonated, according to previous experimental and theoretical study^18^,^55^,^56^ The bridging water W301 in crystal structure is included in the initial model. The structures of the inhibitors are optimized at HF/6–31G** level and their atomic charges are obtained by fitting to the electrostatic potential (ESP) using the restrained ESP (RESP) method^57^ at B3LYP/cc-PVTZ level. The general AMBER force field (GAFF) is adopted to obtain the force field parameters of inhibitors. AMBER12SB force field is employed to produce the parameters of the protein and the crystal water W301. All missing hydrogen atoms are automatically added using the Leap module in AMBER12. Each complex is placed in a truncated periodic box of TIP3P water. The distance from the edges of the box to the closet atoms of the solutes is set to 12 Å. The systems are neutralized by adding chloride counterions. Then they are relaxed in two stages to remove bad contacts between the solute and solvent water molecules. Firstly, only the solvent molecules are optimized by holding the solute fixed with an external force. Secondly, the whole system is energy-minimized without constraint until convergence is reached. After that, the entire systems are heated from 0 to 300 K in 300 ps, followed by 700 ns MD simulation without any restrains at a constant temperature of 300 K with a time step of 2fs. A cutoff of 12 Å is used for the non-bonded interactions and the long-range electrostatic interactions are calculated by the particle mesh Ewald (PME) method^58^. The temperature is regulated by the Langevin dynamics^59^ with a collision frequency of 1.0 ps^−1^. And the SHAKE algorithm^60^ is employed to constrain all bonds involving hydrogen atoms.

PPC is fitted to electrostatic potentials by a combination of the recently developed molecular fractionation with conjugate caps (MFCC)^33^ approach and Poisson-Boltzmann solvation model using an iterative approach as detailed described in ref. 29. A brief description of fitting atomic charge is as follows: the entire protease is firstly decomposed into amino acid based fragment molecules using MFCC scheme to achieve the electronic density distribution of fragment molecules and ligand at B3LYP/6–31 G* level. In the quantum mechanical calculation of each fragment, other residues and ligand are taken as background charges and when calculating the ligand, the total protein is taken as background charges to include the intraprotein polarization effect. Subsequently, the RESP^57^ program is used to generate the partial charge of each atom and ligand according to the electron density of each fragmental molecule. Then Poisson-Boltzmann equation is solved the self-consistent reaction field equation to generate the induced charges on the solute-solvent interface. The induced charges which are applied to mimic the solvation effect are then taken as additional background charges into the next cycle quantum mechanical calculation to fit new atomic charges. The newly calculated atomic charges of solute are used again to calculate new solvent induced charges and these steps are repeated until the atomic charges and induced charges converge. Finally, a new set of charges of protease and ligand are obtained and they correctly represent the polarized condition of each amino acid and ligand embedded in a unique electrostatic environment in the solvated complex. When preforming MD simulation using PPC, the solute charges are simply replaced by the PPC while keeping other parameters of AMBER12SB intact. In this report, the protease, ligand, and the bridging water W301 which is taken as other ligand are treated as complex in charge fitting in PPC scheme. However, we only fit the atomic charges of protease and ligand, and W301 is not included in the complex in the fitting procedure in PPC1 scheme.

The binding free energy of protein-inhibitor is evaluated using MM/PBSA (Molecular Mechanics/Poisson-Boltzmann Surface Area) program^61^,^62^,^63^ in AMBER12. 1000 snapshots are extracted along MD trajectory after equilibrium for the analysis of the binding free energy between six inhibitors and PR. The binding energy (ΔG) in condensed phase can be simply defined by the following equations^64^,^65^.


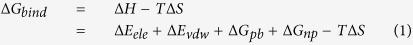



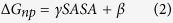


Where Δ*E*_*ele*_ and Δ*E*_*vdw*_ stand for the contribution of electrostatic and van der Waals (vdW) contributions to the binding energy. Δ*G*_*pb*_ and Δ*G*_*np*_ represent the polar and non-polar solvation free energy terms. Δ*G*_*pb*_ is obtained by solving PB equation using the PBSA module of the AMBER suite. The interior and exterior dielectric constants are set to 1 and 80 respectively. Δ*G*_*np*_ is calculated by the solvent-accessible surface area (SASA) using the MSMS program according to the eq. (2)^66^. The surface tension γ and the offset β are the standard values for 0.00542 kcal/(mol · Å2) and 0.92 kcal/mol, respectively. Entropy contribution (*T*Δ*S*) is calculated from normal mode approximation using the NMODE module^67^. Due to these calculations for large systems being extremely time consuming, only 100 snapshots of the 1000 snapshots for each MD trajectories are applied to the entropy calculation. Especially, the W301 is considered as part of PR in the calculation of binding affinities between PR and inhibitor.

## Results and Discussion

### Stability of bridging water W301

In this present work, MD simulations are carried out for 500 ns/700 ns/700 ns, 500 ns/700 ns/700 ns, 500 ns/500 ns/700 ns, 500 ns/700 ns/700 ns, 700 ns/500 ns/700 ns and 500 ns/500 ns/700 ns using AMBER, PPC1 and PPC methods for the six complex (1EBY, 1EC0, 1W5V, 1W5W, 1W5X and 1W5Y) respectively. The dynamics simulation shows that the bridging water W301 drifts away from the binding pocket in the standard AMBER simulation. And W301 begins to leave at around 35 ns, 26 ns, 18 ns, 250 ns, 630 ns and 330 ns, respectively as shown in Fig. 2 which plots the time evolution of the hydrogen bond between W301 and six inhibitors under three force fields. However, in PPC1 simulation, W301 is still far away from the binding pocket in the three of six systems at about 300 ns, 350 ns, 350 ns for 1W5V, 1W5X, 1W5Y respectively. As seen clearly in this figure, in all six systems, the same hydrogen bond is well preserved suggesting the W301 tightly bound to its original position under PPC simulation. This can be explained that the polarized hydrogen bond interacts to the W301, which lengthens its stability in the binding pocket. Due to the W301 is found to drift away from the binding pocket in 500 ns in some simulations of AMBER and PPC1, these MD simulations are not need to be performed for up to 700 ns. So it can be seem from Fig. 2, the MD trajectories with AMBER and PPC1 force field are not as long as that of PPC. The result demonstrates that correctly including the electronic polarization effect in MD simulation is of critical importance in helping stabilize the bridging water molecule and therefore plays indispensable roles in PR and inhibitor interaction.

### Stability of hydrogen bonds of intra-PR, PR-inhibitor

**Figure 1:**
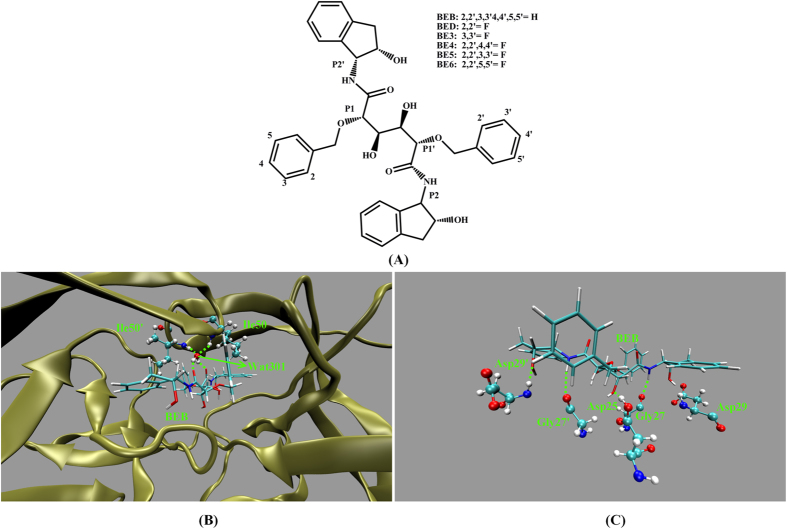
(**A**) Molecular structures of six inhibitors (**B**) The initial structure of PR-BEB complex. The hydrogen bonds formed between W301 and residues (Ile50/Ile50′)/BEB shown as dashed lines. (**C**) The hydrogen bonds formed between BEB and ASP25, GLY27/GLY27′, ASP29/ASP29′ in PR.

**Figure 2:**
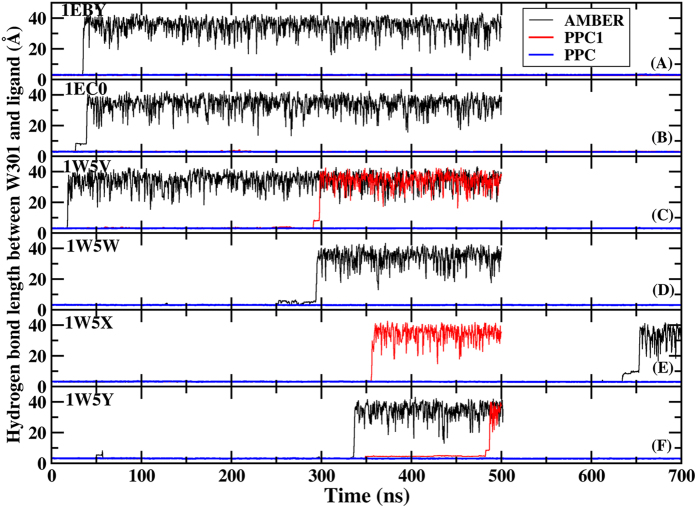
The time evolution of the hydrogen bond between W301 and six inhibitors under AMBER (black), PPC1 (red) and PPC (blue) force fields respectively.

**Figure 3:**
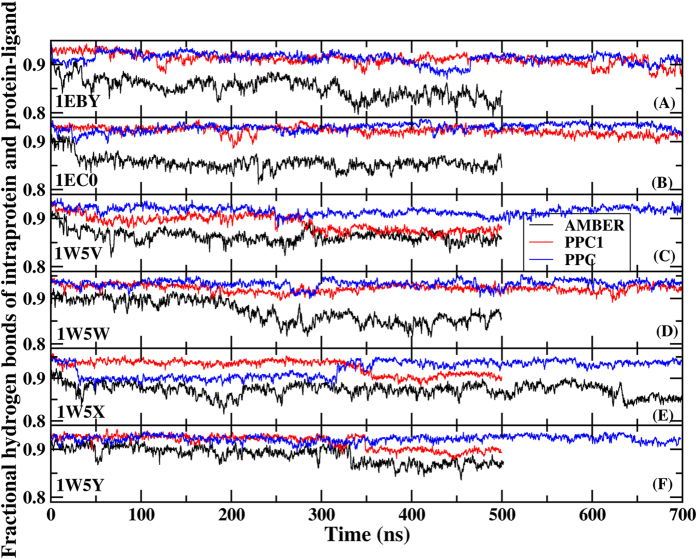
The time evolution of the fractional native hydrogen bonds in intra-PR and PR-inhibitor for six systems under AMBER (black), PPC1 (red) and PPC (blue) force fields respectively.

**Figure 4:**
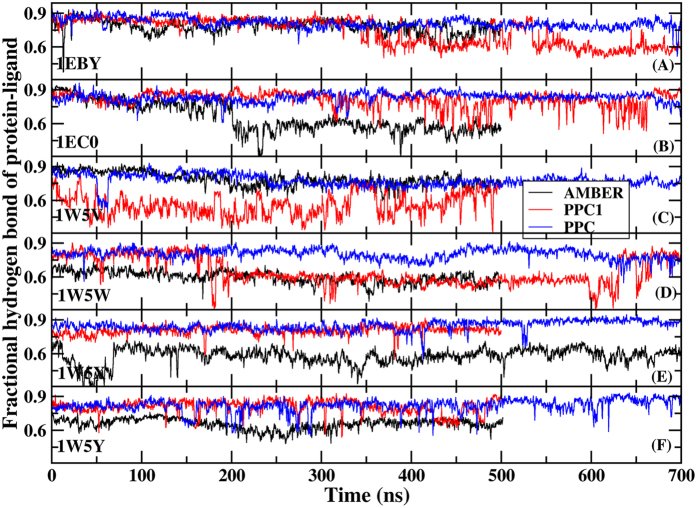
The time evolution of the fractional native hydrogen bonds in PR-inhibitor for six systems under AMBER (black), PPC1 (red) and PPC (blue) force fields respectively.

**Figure 5:**
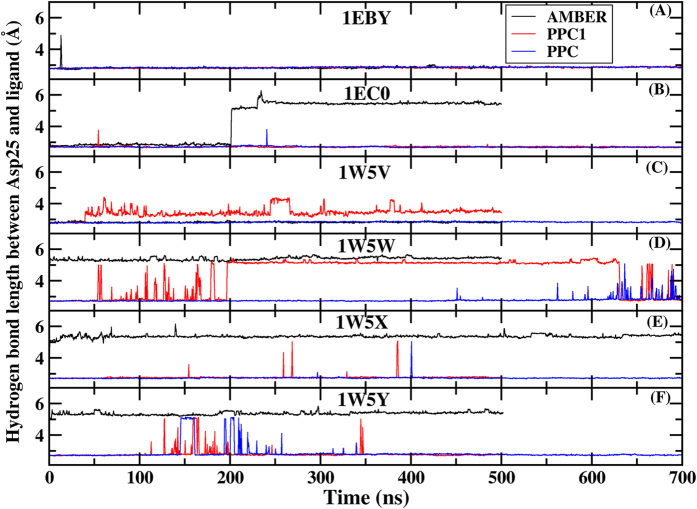
The time evolution of the hydrogen bond between ASP25 and six inhibitors under AMBER (black), PPC1 (red) and PPC (blue) force fields respectively.

To further understand the influence of W301 on the complex structure and the interaction between PR and inhibitor, we study the time dependence of the fractional native hydrogen bonds in intra-PR and PR-inhibitor (shown in Fig. 3) as a function of MD simulation time for six systems using three force fields respectively. A hydrogen bond is counted if the distance between two heavy atoms (N and O in this case) is less than 3.5 Å and the angle N-H-O is large than 120 degree. Fractional number of hydrogen bonds is the number of native backbone hydrogen bonds in PR and PR-inhibitor presented in both the X-ray and the simulation structures divided by the total in the X-ray structure. Obviously, among the three methods, the fractional number of hydrogen bonds is the highest in PPC simulation fluctuated around 95% in the six systems as shown in Fig. 3 (by the blue curves), and following is in PPC1 simulation. However, fractional number of hydrogen bonds formed in AMBER significantly reduces from the initial 100% and continuously fluctuates about 85% afterwards in those systems. The result shows that more native hydrogen bonds are preserved in PPC simulation than in AMBER simulation. Then, we investigate the time evolution of hydrogen bonds formed between PR and inhibitors. Six inhibitors can form five common hydrogen bonds with residues ASP25, GLY27/GLY27′, ASP29/ASP29′ in PR respectively shown in Fig. 1(C). The fractional hydrogen bonds of PR-inhibitors over the course of simulation are also illustrated in Fig. 4. Similar phenomenon is observed that the highest and most stable fractional hydrogen bonds are from PPC simulation and following is in PPC1 simulation with the equal or lower fraction compared to PPC. And the least is in AMBER simulation. We also calculate the length of individual hydrogen bonds formed in PR-inhibitors and the time evolution of specific hydrogen bonds between ASP25 and inhibitor are plotted in Fig. 5 in three force fields for six systems during MD simulation. The hydrogen bonds are very stable with the length of hydrogen bond in each of these six proteins remaining close to the value in native structure during PPC simulation, indicating the hydrogen bonds are well preserved. However, the corresponding hydrogen bond in 1W5W, 1W5X, 1W5Y systems breaks rather quickly at the beginning of AMBER simulation, and in 1EC0 system, it breaks after about 200 ns simulation time, as seen clearly in Fig. 5. On the other hand, under PPC1 force field, the hydrogen bond in 1W5V system undergoes large fluctuation after about 40 ns simulation. For 1W5W system, the hydrogen bond breaks after 50 ns simulation. Without both the stable W301 and the proper electronic polarizable in PPC1 and AMBER force field result into that the hydrogen bonds of intramolecular and intermolecular could not be accurately described from MD simulation.

### Stability of the inhibitor structure

**Figure 6:**
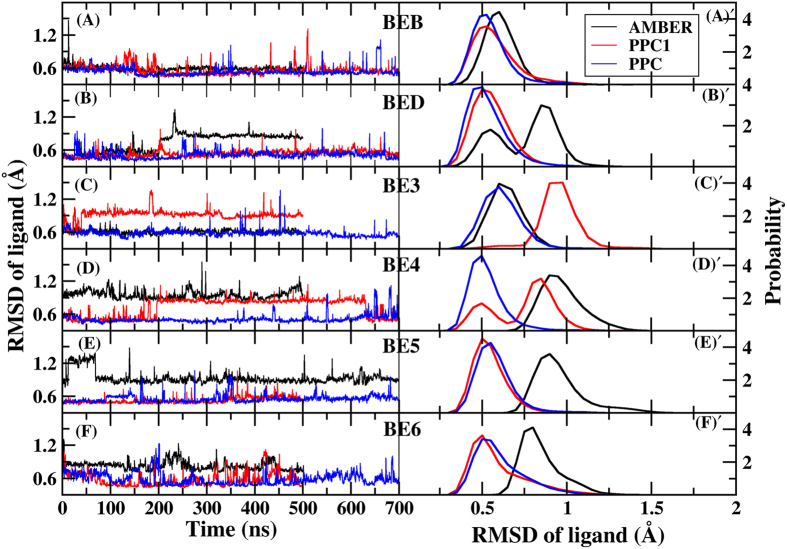
The time evolution of RMSDs for six inhibitors relative to the initial structures (left) and their distributions (right) under AMBER (black), PPC1 (red) and PPC (blue) force fields respectively.

We further investigate the influence of the breaking PR-inhibitors hydrogen bonds on inhibitor structures, so the RMSD values for six inhibitors relative to the initial structures and their distributions during three MD simulations are calculated and depicted in Fig. 6. It is apparent that those inhibitors undergo much less amplitude of vacillation under PPC force field compared to the other two force fields and AMBER simulation shows the most fluctuation besides BE3 inhibitor. The most populated states (shown on the right in Fig. 6) have a RMSD of 0.60/0.53/0.53, 0.85/0.50/0.50, 0.60/0.91/0.60, 0.90/0.85/0.50, 0.90/0.50/0.50 and 0.81/0.50/0.50 Å for six inhibitors using AMBER, PPC1 and PPC respectively, in which PPC has the lowest RMSD. This result is not too surprising in view of that PPC has the best performance. Since W301 is far away from the binding pocket under AMBER and PPC1 simulations, this results in some intra-protein and protein-inhibitor hydrogen bonds broken and the breaking of hydrogen bonds is responsible for the structural incorrect deformation.

### Total binding free energies

**Figure 7:**
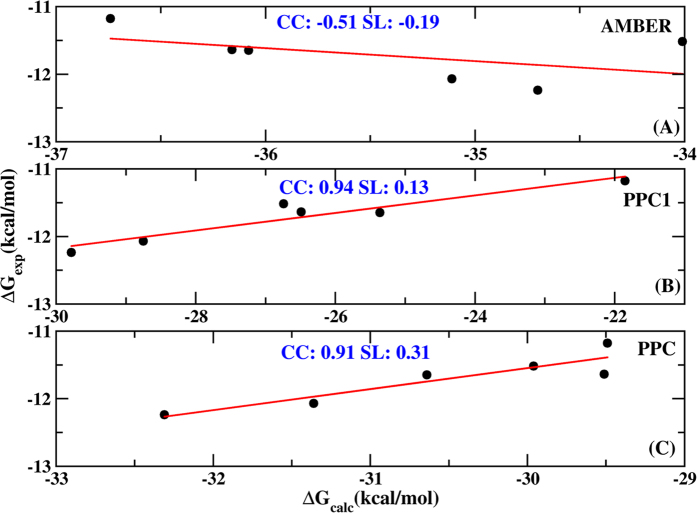
Correlations between the calculated (Δ*G*_*calc*_) and experimental (Δ*G*_exp_) binding free energies under AMBER (**A**), PPC1 (**B**) and PPC (**C**) force fields respectively. CC denotes the correlation coefficient and SL denotes the slope of the line.

The absolute binding free energies are calculated for all six systems using the MM/PBSA method under the three difference simulations. Those snapshots are extracted before W301 leaves the binding pocket under AMBER and PPC1 simulations for direct comparison with the results obtained from PPC simulation. The total binding free energies of the six systems from three simulations are summarized in the Table 1 and the correlations between the calculated and experimental binding free energies are illustrated in Fig. 7. A good correlation is obtained with the correlation coefficient 0.91 and the slope of the line 0.31 in PPC simulation. Furthermore, it is encouraging that the rank of the predicted binding free energies almost agrees with the experimental rank. This implies that the MM/PBSA analysis with the polarized charge can give reliable and reasonable binding affinities. Although PPC1 simulation also shows good correlation with the coefficient 0.94, the credibility of the result is uncertain based on the aforementioned analysis. For comparison, AMBER simulation gives a much weaker correlation and the correlation coefficient is found to be −0.51 due to the lacking of polarization effect. So, all analyses hereafter will based on the PPC simulation. From Table 1, the best strongest binding free energy (−32.31 kcal/mol) comes from 1EBY system, the second is from 1W5W and then follows 1EC0. 1W5V, 1W5X and 1W5Y systems show similar binding affinities. Compared with the 1EBY, the binding strength decreases by 1.67, 2.82, 0.95, 2.35, 2.80 kcal/mol in other five systems respectively, which indicates that the fluoro-substitutions inhibitors produce weaker bindings to PR than nonsubstituted inhibitor. This also suggests that they have the low binding efficacy which is in good agreement with the experimental observations^52^.

**Figure 8:**
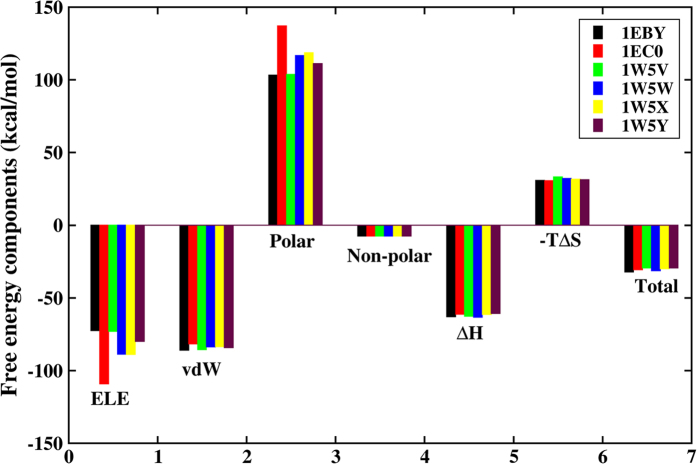
Contributions of the binding free energy components of six inhibitors to PR based on PPC simulation respectively.

Contributions of the binding free energy components are shown in Fig. 8 and Table 2 based on PPC simulation. According to Table 2, the correlation coefficients between the experimental affinities and the binding free energy components are −0.03, 0.11, 0.05, 0.07, 0.44 and 0.57 for the electrostatic energy, van der Waals energy, polar solvation energy, nonpolar solvation energy, enthalpy change and the entropy compensation, respectively. Despite each term gives poor linear relationship, the sum of them clearly improves the quality of the fit and the correlation coefficient between the total binding free energy and experiment value is 0.91, which implies that the binding free energy arises from a very complicated interaction in all of these components. As seen from Table 2 and Fig. 8, in all six PR-inhibitor complexes, electrostatic and van der Waals terms in the gas phase are the basis for favorable binding free energies. However, the polar solvation energies produce the unfavorable contributions to the binding affinities which offset the favorable electrostatic terms. Finally, the results of balancing the two terms contributions are unfavorable for the binding free energies for six systems. The non-polar solvation energies corresponding to the burial of SASA contribute slightly favorably on the binding, whereas entropy terms oppose the inhibitors binding. It is noticeable that van der Waals energies are almost 11 times stronger than the non-polar solvation energy in all complexes. So, the van der Waals terms are the main driving force of inhibitor binding with the largest component of the binding free energies for each system ranging from −86 to −82 kcal/mol.

### Decomposition of binding free energy

**Figure 9:**
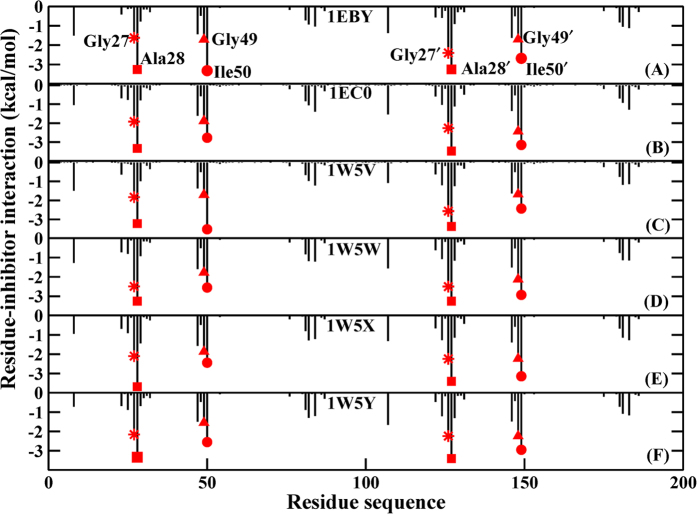
Decomposition of the binding free energy on a per-residue basis for the PR-inhibitors complexes based on PPC simulation respectively.

**Figure 10:**
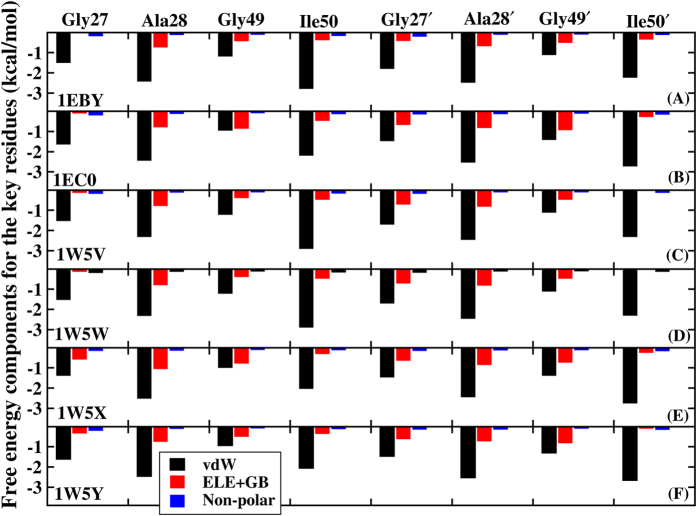
Decomposition of the binding free energy on a per-residue basis into contributions from the vad der Waals (vdW) energy, the sum of electrostatic energy and polar solvation energy, and non-polar solvation energy for the key residues in six systems based on PPC simulation respectively.

**Figure 11:**
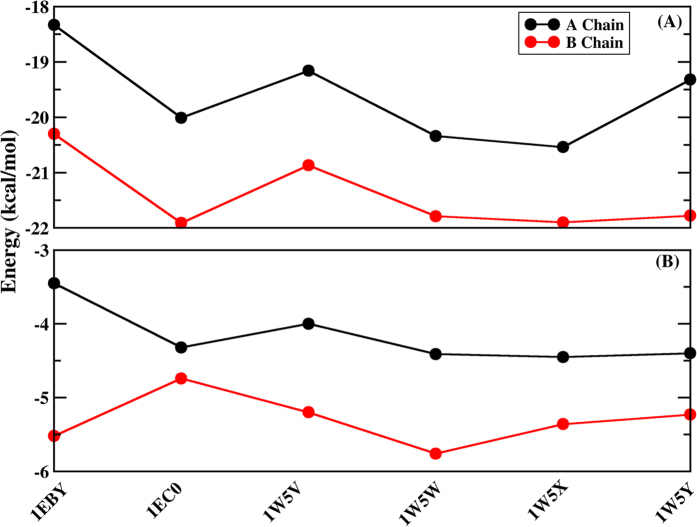
(**A**) The binding free energy between inhibitors and A chain (black), inhibitors and B chain (red) of PR for six systems based PPC simulation respectively. (**B**) The binding free energy between W301 and A chain (black), W301 and B chain (red) of PR for six systems based PPC simulation respectively.

In order to gain more-detailed investigation for the contributions of each residue to binding free energy, the binding free energy is decomposed into inhibitor-residue pairs and their interaction spectrum are plotted in Fig. 9. This is extremely useful to understand the PR-inhibitor interaction mechanism at atomic level. Overall, the interaction spectrums are quite similar in six systems and the dominant favorable interactions come from the regions of catalytic site (GLY27/GLY27′, ALA28/ALA28′ residues) and flap (GLY49/GLY49′, ILE50/ILE50′ residues). Among them, ALA28/ALA28′ and ILE50/ILE50′ residues with inhibitor produce the interaction larger than 2.4 kcal/mol. GLY27/GLY27′, GLY49/GLY49′ residues have also major binding attractions with inhibitor with the energy over 1.6 kcal/mol. Again, the decomposition of binding free energy on a per-residues basis into contributions of van der Waals, the sum of electrostatic term in the gas phase and polar solvation energy and non-polar solvation energy for the above eight residues in six complexes are shown in Fig. 10. It is worthwhile that the dominant favorable interactions come from van der Waals interaction with the energies in the range of −1.0 to −2.9 kcal/mol for the eight residues and other two terms contribute slightly to the binding free energy. In addition, one observation from Figs 9 and 10 shows that the binding modes of these inhibitors to A chain are similar to B chain of PR, but they tend to have slightly stronger interaction with B chain than that with A chain (Fig. 11A). The small differences of energy between B chain and A chain are −1.97, −1.90, −1.71, −1.45, −1.36, −2.46 kcal/mol for the six complexes respectively. Among all systems, those inhibitors have stronger electrostatic interaction with deprotonated ASP25′ through charge-dipole interaction than the protonated ASP25 for about 0.3~1.2 kcal/mol. Moreover, for 1EBY, 1W5V systems, the binding affinity of the GLY27 to inhibitor decreases by 0.91, 0.73 kcal/mol compared to the GLY27′ respectively resulting in the main difference of energy. This is likely because that GLY27 forms weaker 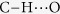 hydrogen bond (slightly lower occupancy) with inhibitor than GLY27′. For 1EC0, 1W5W, 1W5X, and 1W5Y systems, the energy differences of A and B chain are dominated by the binding differences of GLY49 and GLY49′, ILE50 and ILE50′, which account for 49%, 50%, 78% and 44% of the total differences of the binding affinity respectively. Study finds that GLY49 and ILE50 in A chain have weaker van der Waals interactions with inhibitor than those in B chain. The decreases in the van der Waals interactions between GLY49, ILE50 and inhibitor provide the main contributions to the energy differences. The relative binding free energy differences from above calculations might be caused by the structures of those inhibitors.

### PR-inhibitor hydrogen bond

**Figure 12:**
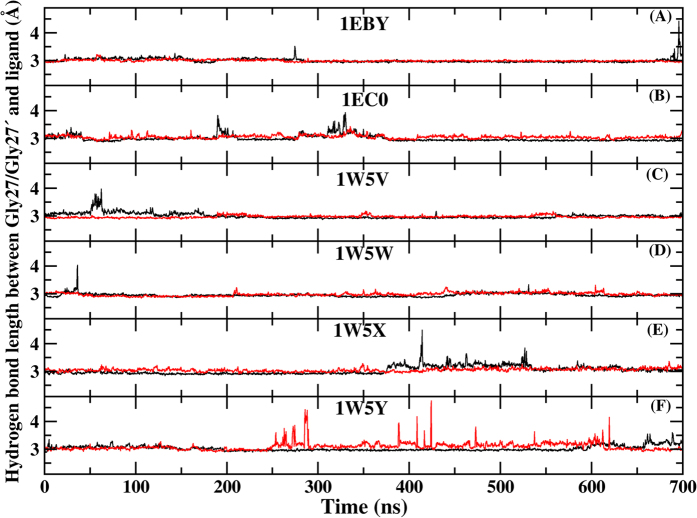
The time evolution of the hydrogen bond between GLY27 (black)/GLY27′(red) and six inhibitors based PPC simulation respectively.

The hydrogen bonds between PR and inhibitor are essential for the binding affinity, which can be indirectly surveyed by the occupancy. There are five common hydrogen bonds formed between inhibitor and residues ASP25, GLY27/GLY27′, ASP29/ASP29′ of PR together and their occupancies are listed in Table 3 in six systems. According to Table 3, the occupancies of the three hydrogen bonds formed residues ASP25, GLY27/GLY27′ with inhibitor are higher than 90% which suggests that fluoro-substituted inhibitors do not produce large influence on the stability of above three hydrogen bonds. And the time evolutions of hydrogen bond lengths between GLY27 and six inhibitors, GLY27′ and six inhibitors during MD simulation are shown in Fig. 12. These two hydrogen bonds are very stable fluctuating around 3 Å. Although these hydrogen bonds are instantaneously broken for some time, they quickly reform and then remain stable in the remaining time. The hydrogen bonds formed between ASP29/ASP29′ and inhibitor are instable with the occupancies under 75% for all six systems. Compared with other five systems, the occupancy of hydrogen bond formed between residues ASP25 and inhibitor for 96% is slightly decreased in 1W5Y. The occupancies of GLY27/GLY27′-inhibitor hydrogen bonds are also decreased in 1EC0, 1W5X and 1W5Y systems compared to those in the 1EBY, 1W5V, 1W5W systems. For the hydrogen bond formed between ASP29 and inhibitor, the occupancies in 1EC0, 1W5V and 1W5W systems are similar to those in the 1EBY system, while 1W5X, 1W5Y significantly increases the occupancy of the hydrogen bond. The stability of ASP29′-inhibitor hydrogen bond, as reflected in the occupancy, is relatively low in all systems except 1EC0. On the basis of the above analyses, three strongest (most occupied) hydrogen bonds form between inhibitor and the three residues ASP25, GLY27/GLY27′ located the catalytic site and they are present during >90% of the simulation in all systems. The result suggests that the three hydrogen bonds play an important role in maintaining the binding of the inhibitor to PR after the fluoro-substitutions.

### Contribution of W301 to the binding free energy

In six complexes, W301 bridges the PR and inhibitors by accepting two hydrogen bonds from the NH group of ILE50/ILE50′ and donating two hydrogen bonds to the carbonyl groups at those inhibitors. Due to the importance, the contribution of W301 to the binding free energy is estimated by employing MM/PBSA method. And the results are shown in Table 4. The ratios of the binding free energies between W301 and PR/inhibitors to total binding free energy between PR and inhibitors are 4.43%, 5.52%, 9.26%, 14.54%, 11.75% and 14.94% respectively in the six systems. Current study indicates that the W301 contributes significantly to the binding free energy of PR/inhibitors complex. As can be seen in Table 3, W301 of 1W5W system produces the strongest binding free energy (−4.56 kcal/mol) to PR/inhibitor followed by 1W5Y, 1W5X, 1W5V, 1EC0. And the weakest binding free energy (−1.43 kcal/mol) comes from the 1EBY system among the six systems which suggests that difluoro-substituted inhibitors improve the binding affinities between W301 and PR/inhibitors. The binding of W301 to the PR/inhibitor is dominated by the hydrogen bond interactions with ILE50/ILE50′ and inhibitor. Further analysis finds that W301 forms relatively weak hydrogen bond with ILE50 of A chain in 1BEY with the occupancy of 94% which results in the decrease of binding affinities. Figure 11(B) displays the comparison of binding free energy between W301 and two chains of PR. Obviously, the binding affinity from W301 with B chain to W301 with A chain is decreased by −2.07 kcal/mol in 1EBY system due to the slightly weak hydrogen bonds formed between W301 and A chain. In addition, W301 also have a slightly stronger interaction with B chain than that with A chain of protease in other five systems. Although those hydrogen bonds formed between W301 and two chains are all very stable with the occupancies over 97% in the five systems, W301 is found to form stronger electrostatic interaction with GLY49′ in B chain than that in A chain.

## Conclusion

This work provides a quantum mechanical prediction of the binding free energy in PR and six inhibitors using quantum-based polarized protein-specific charge (PPC) method. Our study shows intra-PR and PR-inhibitor hydrogen bonds, complexes structures, especially the bridging water W301, are stabilized by polarization and the calculated binding free energies have a significantly linear correlation with experimental results with the correlation coefficient of 0.91. However, W301 is found to be far away from the binding pocket through the use of the conventional AMBER force field at the same simulation time as PPC. And AMBER simulation gives poor linear relationship between the theoretical and experimental binding free energies with the correlation coefficient of −0.51 due to the lack of polarization effect. This study emphasizes that the importance of incorporating polarization effect into MD simulation and free energy calculation to precisely predict the binding affinity.

Furthermore, our study based on PPC method reveals that the interaction spectrums of six inhibitors with PR are quite similar and dominant favorable interactions come from van der Waals interaction between GLY27/GLY27′, ALA28/ALA28′ GLY49/GLY49′, ILE50/ILE50′ and inhibitors. And the three hydrogen bonds form between residues ASP25, GLY27/GLY27′ and inhibitors are quite stable in all systems and therefore play an important role in maintaining the binding of the inhibitor to PR. And these inhibitors are found to have stronger interaction with B chain than A chain. Also, our result shows that W301 contributes significantly to the binding free energy of PR/inhibitors complexes emphasizing its importance in the prediction of binding affinity. W301 also have a slightly stronger interaction with B chain than that with A chain of protease. The information obtained from the current studies provides useful insights on the PR-inhibitor binding mechanism. Meanwhile, it will be valuable for the design of new potent inhibitors in the future.

## Additional Information

**How to cite this article**: Duan, L. L. *et al*. Effect of polarization on HIV-1protease and fluoro-substituted inhibitors binding energies by large scale molecular dynamics simulations. *Sci. Rep.* **7**, 42223; doi: 10.1038/srep42223 (2017).

**Publisher's note:** Springer Nature remains neutral with regard to jurisdictional claims in published maps and institutional affiliations.

## Figures and Tables

**Table 1:** Binding free energies (kcal/mol) from MM/PBSA calculation using three simulations.

Complex	AMBER	PPC1	PPC	Exp*
1EBY	−34.70	−29.78	−32.31	−12.24
1EC0	−36.08	−25.36	−30.64	−11.65
1W5V	−36.74	−21.85	−29.49	−11.18
1W5W	−35.11	−28.75	−31.36	−12.07
1W5X	−34.01	−26.74	−29.96	−11.52
1W5Y	−36.16	−26.49	−29.51	−11.64

^*^The experimental binding free energy is calculated using K_i_, which is provided from Jimmy *et al*.^52^

**Table 2:** Binding free energies components for PR-inhibitors complexes based on PPC simulation, Errors labeled by the signs ± represent the standard deviations.

Complex	Δ*E*_*ele*_	Δ*E*_*vdw*_	Δ*G*_*pb*_	Δ*G*_*np*_	Δ*H*	−*T*Δ*S*	Δ*G*_*bind*_
1EBY	−72.55 ± 5.6	−86.09 ± 3.6	103.19 ± 3.5	−7.52 ± 0.1	−62.96 ± 4.7	30.65 ± 5.9	−32.31
1EC0	−109.15 ± 6.5	−81.64 ± 4.2	137.01 ± 4.2	−7.44 ± 0.1	−61.22 ± 5.2	30.58 ± 6.4	−30.64
1W5V	−73.07 ± 6.7	−85.61 ± 4.0	103.60 ± 4.1	−7.54 ± 0.1	−62.61 ± 5.0	33.12 ± 5.5	−29.49
1W5W	−88.83 ± 6.7	−83.69 ± 3.9	116.65 ± 4.4	−7.52 ± 0.1	−63.39 ± 4.8	32.03 ± 6.5	−31.36
1W5X	−88.95 ± 7.1	−83.58 ± 4.2	118.51 ± 4.6	−7.49 ± 0.1	−61.51 ± 4.9	31.55 ± 6.4	−29.96
1W5Y	−79.94 ± 6.3	−84.35 ± 3.8	111.06 ± 4.4	−7.45 ± 0.1	−60.69 ± 4.7	31.18 ± 6.2	−29.51

**Table 3:** Hydrogen bonds formed between PR and inhibitor based on PPC simulation.

Hydrogen Bond	Occupancy (%)					
	1EBY	1EC0	1W5V	1W5W	1W5X	1W5Y
ASP25-inhibitor	99.90	99.88	99.96	98.80	99.76	96.14
GLY27-inhibitor	98.37	96.75	97.68	98.67	92.83	96.24
ASP29-inhibitor	57.65	51.53	50.95	64.02	72.46	71.41
GLY27′-inhibitor	99.08	96.96	99.02	98.77	96.76	92.50
ASP29′-inhibitor	50.59	70.44	40.45	41.37	61.56	52.83

Occupancy is defined as the percentage of simulation time in which a specific hydrogen bond exists.

**Table 4:** Binding free energies components for PR/inhibitors and W301 based on PPC simulation, Errors labeled by the signs ± represent the standard deviations.

Complex	Δ*E*_*ele*_	Δ*E*_*vdw*_	Δ*G*_*pb*_	Δ*G*_*np*_	Δ*H*	*−T*Δ*S*	Δ*G*_*bind*_	_Ratio_
1EBY	−34.20 ± 3.1	0.76 ± 1.7	23.15 ± 0.9	−0.75 ± 0.0	−11.04 ± 2.9	9.61 ± 3.2	−1.43	4.43%
1EC0	−31.90 ± 3.2	1.53 ± 2.0	20.14 ± 0.3	−075 ± 0.0	−10.97 ± 3.0	9.28 ± 4.2	−1.69	5.52%
1W5V	−36.43 ± 3.5	0.21 ± 1.8	25.18 ± 1.2	−0.75 ± 0.0	−11.78 ± 3.0	9.05 ± 4.0	−2.73	9.26%
1W5W	−38.22 ± 3.1	0.27 ± 1.6	25.83 ± 1.2	−0.75 ± 0.0	−12.86 ± 2.7	8.30 ± 3.6	−4.56	14.54%
1W5X	−35.19 ± 3.0	0.20 ± 1.7	24.05 ± 1.0	−0.75 ± 0.0	−11.68 ± 2.7	8.16 ± 4.0	−3.52	11.75%
1W5Y	−37.39 ± 2.9	0.46 ± 1.7	25.72 ± 1.1	−0.74 ± 0.0	−11.95 ± 2.6	7.54 ± 3.6	−4.41	14.94%

Ratio denotes the ration of binding free energy contribution of W301to the total binding free energy.
